# Emergency medical services transport delays for suspected stroke and myocardial infarction patients

**DOI:** 10.1186/s12873-015-0060-3

**Published:** 2015-12-03

**Authors:** Ashley Pedigo Golden, Agricola Odoi

**Affiliations:** Department of Biomedical and Diagnostic Sciences, The University of Tennessee, 2407 River Drive, Knoxville, TN 37996-4543 USA

**Keywords:** Myocardial infarction, Stroke, Disparities, Emergency medical services, EMS, Transport times, Prehospital delays

## Abstract

**Background:**

Prehospital delays in receiving emergency care for suspected stroke and myocardial infarction (MI) patients have significant impacts on health outcomes. Use of Emergency Medical Services (EMS) has been shown to reduce these delays. However, disparities in EMS transport delays are thought to exist. Therefore the objective of this study was to investigate and identify disparities in EMS transport times for suspected stroke and MI patients.

**Methods:**

Over 3,900 records of suspected stroke and MI patients, reported during 2006–2009, were obtained from two EMS agencies (EMS 1 & EMS 2) in Tennessee. Summary statistics of transport time intervals were computed. Multivariable logistic models were used to identify predictors of time intervals exceeding EMS guidelines.

**Results:**

Only 66 and 10 % of suspected stroke patients were taken to stroke centers by EMS 1 and 2, respectively. Most (80–83 %) emergency calls had response times within the recommended 10 min. However, over 1/3 of the calls had on-scene times exceeding the recommended 15 min. Predictors of time intervals exceeding EMS guidelines were EMS agency, patient age, season and whether or not patients were taken to a specialty center. The odds of total transport time exceeding EMS guidelines were significantly lower for patients not taken to specialty centers. Noteworthy was the 72 % lower odds of total time exceeding guidelines for stroke patients served by EMS 1 compared to those served by EMS 2. Additionally, for every decade increase in age of the patient, the odds of on-scene time exceeding guidelines increased by 15 and 19 % for stroke and MI patients, respectively.

**Conclusion:**

In this study, prehospital delays, as measured by total transport time exceeding guideline was influenced by season, EMS agency responsible, patient age and whether or not the patient is transported to a specialty center. The magnitude of the delays associated with some of the factors are large enough to be clinically important although others, though statistically significant, may not be large enough to be clinically important. These findings should be useful for guiding future studies and local health initiatives that seek to reduce disparities in prehospital delays so as to improve health services and outcomes for stroke and MI patients.

## Background

Despite recent declines in death rates from stroke and acute myocardial infarction (MI), the burdens of these conditions remain high [1]. Both conditions require time sensitive treatments so transport time to appropriate heath facilities is critical. Improved outcomes have been observed for ischemic stroke patients when intravenous thrombolytic treatment is received within 3 h of the onset of symptoms [2]. To ensure timely receipt of treatment, it is recommended that stroke patients be transported directly to accredited stroke centers [2]. For MI, current guidelines recommend that the time from first medical contact to percutaneous coronary intervention (PCI) be 90 min or less [3]. Health outcomes are improved by up to 50 % when PCI is administered within 60 min and by 23 % if given within 180 min of the onset of symptoms [4, 5].

While there are two general types of delays (prehospital and in-hospital) that affect timely receipt of stroke and MI treatments, some studies have suggested that the prehospital interval, from onset of symptoms to arrival at the hospital, is the source of the longest delay [6]. Utilization of Emergency Medical Services (EMS), among other factors, are associated with reduced delays for treatments of stroke [6] and MI [4]. However, most past studies considered prehospital time (from the onset of symptoms to arrival at the hospital interval) as one time interval. To better identify disparities and target interventions, prehospital delays should be further sub-divided into decision delays and transport delays [6]. Since EMS play a critical role in providing rapid transport of acute stroke and MI patients, it is important to investigate the specific time intervals involved: response time, on-scene time, and travel time to the hospital. Unfortunately, only a few recent studies have reported the specific time intervals associated with EMS transport for stroke [7, 8] and MI [9]. Thus, additional studies of different populations, geographic areas, and EMS agencies are needed to improve our understanding of this component of prehospital delays. We hypothesize that there are geographic disparities in transport times for suspected stroke and MI cases. If these disparities are identified, it would help guide efforts to improve transport times for suspected stroke and MI patients Therefore, the objective of this study was to identify and describe disparities in EMS transport time delays for suspected stroke and MI patients.

## Methods

This study was approved by the Institutional Review Board of the University of Tennessee.

### Study population

This study was performed in Tennessee, an area with some of highest mortality risks of stroke and MI in the country [1]. The study included populations living in two counties each served by one of the two EMS agencies that participated in the study. Although other private ambulance services exist in the study area, the two EMS agencies that participated in the study are the largest agencies and respond to the great majority of 911 calls in the study area. One EMS (EMS 1) provided service to a mostly urban county, while the other, EMS 2, served a rural county within a mountainous area. The rural–urban designation is based on US census bureau classification. There were two stroke specialty centers and six cardiac specialty centers located in the urban county served by EMS 1. The county served by EMS 2 had no stroke centers and one cardiac center. Additionally, the areas served by the two EMS agencies had populations with significantly different socio-demographic and other characteristics. For instance, areas served by EMS 2 had significantly higher proportions of individuals whose main language spoken at home was not English as well as those who were 65 years or older compared to areas served by EMS 1. On the other hand, areas served by EMS 1 had significantly higher per capita income and proportions of individuals with at least high school education (Table 1).

**Table 1:** Socio-demographic and other characteristics of the populations served by EMS 1 and EMS 2

Characteristics	EMS 1	EMS 2
Population	432,000	90,000
^a^Persons 65 years and over	14 %	17 %
^a^High School graduate or higher	90 %	82 %
^a^Language other than English spoken at home	6.3 %	6.8 %
^a^Per capita income in past 12 months	$28,000	$22,000
^b^Mountainous (Yes/No)	No	Yes
^b^High Tourist Activity	No	Yes

^a^Significantly different at a *p* ≤ 0.05

^b^No statistical comparison performed

### Data collection and management

Suspected stroke and MI cases reported during 2006–2009 were extracted from EMS dispatch databases obtained from the EMS agencies that served the areas covered by the study. Permission to use these data were provided by the responsible EMS agencies. Suspected stroke or MI cases met at least one of the following criteria: 1) the emergency caller mentioned one or more stroke/MI symptom(s) as defined by the AHA [10]; 2) EMS observed one or more of the defined symptoms; 3) EMS had the clinical impression of a suspected MI/stroke. Information for criterion 1 above was obtained from EMS call records (patient care report). The focus of this study was to assess disparities in transport times of suspected (not confirmed) stroke and MI cases. Since a suspected (not confirmed) diagnosis of stroke and MI would be expected to be the initial basis for the priority level an EMS call receives, it was practical to assess the travel times based on the suspected diagnosis and not necessarily the confirmatory diagnosis since the initial suspected diagnosis is one of the factors that guides the decision of EMS personnel as to the priority assigned to a specific call and subsequently their transport decision. Confidential patient data were removed before records were released to investigators.

Data from the two EMS agencies were analyzed separately because it was hypothesized *apriori* that there are geographic differences in transport times in the areas served by the two EMS agencies for two reasons: (a) topographical differences in the areas served by the two EMS agencies. One EMS serves a mainly mountainous area which brings transportation challenges during the winter months when the road conditions in the mountainous areas are especially challenging; (b) the fact that areas served by one EMS agency was mainly urban while the other was mainly rural and we suspected disparities based on urbanicity. Records provided by EMS 1 were manually entered into an electronic database and then checked for accuracy. However, all records from EMS 2 were received as an electronic database. A total of 4,411 records matched the case definitions. Records were excluded (495 cases, 11.2 %) from analyses under the following conditions: 1) patient was <18 years (165 cases, 3.7 %); 2) duplicates (33 cases, 0.75 %); 3) dispatch did not result in transport (48 cases, 1.1 %); or 4) missing all time components (249, 5.6 %).

The EMS transport time was divided into the following time intervals: total time (from dispatch to hospital arrival), response time (from dispatch to arrival on-scene), on-scene time (from arrival on-scene to departure), and travel time (from scene departure to hospital arrival). The time of the emergency/911 call was missing in over half of the records and thus was not used in calculating EMS response. However, for records with 911 call time recorded, the median elapsed time between receiving the call and ambulance dispatch was 1 min and therefore would have negligible impact on our findings.

Other variables of interest in describing disparities in EMS transport times included: patient age and gender, season (winter: December-February; spring: March-May; summer: June-August; fall: September-November), dispatch reason, and whether or not the patient was taken to a specialty center. Stroke specialty centers were identified based on stroke accreditation by the Joint Commission on the Accreditation of Health Organizations (JCAHO) [11], while cardiac specialty centers were those identified from the JCAHO website as providing PCI. Additional variables collected by EMS 2, but not EMS 1 included: residency status in the study area, red lights/sirens (RLS) mode of the ambulance to the scene or to the hospital, and whether EMS or patient/family members made the choice of hospital to which the patient was taken.

### Data analysis

Descriptive analyses were performed in SAS 9.2 [12]. Cases were considered to exceed guidelines if: response time was >10 min [9], on-scene time was >15 min [9], or total time was >60 min [13]. Significant differences in medians of time intervals between important categorical variables were assessed using Wilcoxon rank sum (for binary variables) or Kruskal-Wallis (for variables with multiple categories) tests. Associations between categorical variables and the binary outcomes of exceeding guidelines were investigated using chi-square test, or Fisher’s exact test for low cell counts. For categorical variables that were significantly associated with exceeding a time guideline, differences between categories were assessed using two-sample test of proportions, adjusted for multiple comparisons using Simes method. A number of variables were investigated for potential simple associations with different time intervals and delays.

Adjusted associations between the potential predictors of times exceeding guidelines (total time, response time and on-scene time) for both MI and stroke were investigated using multivariable logistic regression. The models were built using a manual backwards elimination procedure using likelihood ratio test to assess significance of variables and setting significance level at *p* = 0.05. Since EMS was an important factor of interest, it was forced in all the models. Variables were considered important confounders if their removal from the model resulted in a large (greater than 20 %) [14] change in the coefficients of any of the remaining variables in the model. Categorical variables were analyzed as regular dummy variables. The significance in the model of each group of dummy variables belonging to a categorical variable was assessed using a likelihood ratio test. Two-way interaction terms were assessed for significance and significant ones retained in the final models. Goodness-of-fit of the logistic models were assessed using Deviance and Pearson goodness-of-fit statistics as well as model residual diagnostics.

## Results

### Patient characteristics

#### Suspected stroke

There were 1,075 suspected stroke cases for EMS 1. Significantly (*p* = 0.04) more suspected stroke cases occurred in the fall (31.3 %) than spring (21.0 %), summer (21.4 %), or winter (26.3 %). The most common dispatch reason for EMS 1 suspected stroke patients were ‘Cerebrovascular accident (CVA)/Stroke’ (92.5 %). The majority of patients were females (63.7 %) who were significantly (*p* < 0.0001) older (median: 77, IQR:22 (63–85)) than males (median: 69, IQR:25 (56–81)). Significantly (*p* = 0.0001) more suspected stroke patients were taken by EMS 1 to a stroke center (66.3 %) than to a non-stroke center (33.7 %).

A total of 511 suspected stroke cases were reported by EMS 2. Like EMS 1, significantly (*p* = 0.03) more suspected stroke cases occurred in the fall (35.4 %) than spring (14.9 %), summer (29.9 %), or winter (19.8 %). The most common dispatch reasons for EMS 2 suspected stroke patients were ‘Unconscious/Fainting/Syncope’ (21.1 %), followed by ‘Convulsions/Seizures’ (16.8 %), and ‘CVA/Stroke’ (9.6 %). More suspected stroke patients were female (53 %) and the median patient age was 56 (IQR:36 (39–75)). Unlike suspected stroke patients from EMS 1, there was no difference (*p* = 0.09) in median age between genders for EMS 2 patients. Only 56 % of EMS 2 patients were residents of the study area.

The RLS mode was used to the scene in 93 % of EMS 2 suspected stroke calls and for only 33 % of travels to the hospital. Use of RLS to the hospital was significantly (*p* = 0.008) higher when the dispatch reasons were more serious (e.g. unconscious, stroke) compared to other reasons (e.g. headache, dizziness, etc.). The hospital to which the patient was taken was selected by EMS personnel for 80 % of suspected stroke patients, of which the majority (76 %) were taken to the closest facility. When the dispatch reasons were more serious, the hospital choice tended to be made by EMS personnel (*p* = 0.05).

#### Suspected myocardial infarction (MI)

There were 1,754 suspected MI cases for EMS 1 (Table 2). Similar to suspected stroke, significantly (*p* = 0.03) more cases occurred during fall (36 %) than spring (24.7 %), summer (21.7 %), or winter (17.5 %). The majority of patients were females (53.0 %) who were significantly (*p* < 0.0001) older (median: 60, IQR: 31 (46–77)) than males (median: 56, IQR: 22 (47–69)). The most common dispatch reason was ‘Chest pain’ (98.7 %). The majority (96 %) of suspected MI patients were taken to cardiac centers by EMS 1.

**Table 2:** Prehospital transport time intervals for stroke and myocardial infarction patients in East Tennessee

EMS 1					EMS 2				
Time (Minutes)	N	Median	Min, Max	IQR^a^	N	Median	Min, Max	IQR^a^	*p* ^+^
Stroke	1075				511				
Total	1041	35	(4, 78)	13 (28.5–41.5)	509	41	(<1, 144)	18 (32–50)	<.0001
Response	1047	6	(<1, 45)	5 (3.5–8.5)	506	4	(<1, 46)	5 (1.5–6.5)	<.0001
On-scene	1052	13	(<1, 39)	8 (9–17)	509	14	(<1, 122)	9 (9.5–18.5)	0.093
Travel	1048	15	(<1, 47)	8 (11–19)	509	23	(<1, 77)	16 (11.5–27.5)	<.0001
MI	1754				576				
Total	1717	34	(3, 97)	14 (27–41)	572	41	(<1, 123)	17 (32.5–49.5)	<.0001
Response	1738	6	(<1, 32)	4 (4–8)	573	4	(<1, 27)	5 (1.5–6.5)	<.0001
On-scene	1739	13	(<1, 73)	7 (9.5–16.5)	575	12	(<1, 51)	11 (6.5–17.5)	0.61
Travel	1728	14	(<1, 68)	9 (9.5–18.5)	572	23	(<1, 94)	8 (19–27)	<.0001

*Min* minimum

*Max* maximum

^a^ *IQR* Interquartile range

^+^ *p*-value for difference between median times of EMS 1 & 2

A total of 576 suspected MI cases were recorded by EMS 2. There were no differences in the volume of cases across seasons. The majority of suspected MI patients transported by EMS 2 were females (52 %) who were significantly (*p* < 0.0001) older (median age: 62; IQR: 29 (47–76)) than males (median age: 54; IQR:23 (43–66)). ‘Chest pain’ (51.8 %) and ‘Shortness of breath’ (28.5 %) were the two most common dispatch reasons for EMS 2 patients. Almost all (98.8 %) EMS 2 suspected MI patients were taken to cardiac centers.

Similar to suspected stroke patients for EMS 2, only 59 % of suspected MI patients were study area residents. RLS were used to the scene for 96 % of patients. This was significantly (*p* = 0.04) higher when the dispatch reason was ‘Chest pain’ compared to others. In contrast, RLS were used for 36 % of patients during travel to the hospital. The hospital was selected by EMS 2 personnel for 75 % of suspected MI patients, with the majority (79 %) going to the closest facility. Significantly (*p* = 0.02) more females (61 %) and study area residents (85 %; *p* < 0.0001) had the hospital choice made by patient/family members.

### Transport time intervals

#### Suspected stroke

Summary statistics for the different time intervals for suspected stroke patients are presented in Table 2. The total time was significantly (*p* < 0.0001) longer for EMS 2 patients (41 min) compared to EMS 1 (35 min) (Table 2). Patients served by EMS 2 had significantly (*p* < 0.0001) shorter response times than EMS 1 patients. Patients taken to stroke centers by EMS 1 had significantly shorter (*p* < 0.0001) travel times (median: 15) than those taken to non-stroke centers (median: 20). More serious dispatch reasons were associated with significantly shorter response (median: 5; IQR: 5 (3–8); *p* = 0.02) and travel (median: 21; IQR: 8 (18–26); *p* = 0.001) times compared to the response (median: 6; IQR: 5 (4–9)) and travel (median: 23; IQR: 8 (20–28)) times for less serious dispatch reasons. It is worth noting that some of the differences in time intervals between the two EMS agencies were both statistically significant and large enough to be clinically important while others were statistically significant but might not be large enough to be clinically important. For instance, the absolute difference in total time interval (6 min) and that of travel time interval (8 min) were both statistically significant and large enough to be clinically important while the difference in response time interval (2 min) although statistically significant may not be large enough to be clinically important.

Patients taken to stroke centers by EMS 2 had significantly (*p* = 0.01) longer on-scene times (median: 17; IQR: 9 (12–21)) and shorter travel times (median: 14; IQR: 15 (8–23)) compared to on-scene (median: 13; IQR: 10 (9–19)) and travel (median 23: IQR: 8 (20–28)) times of patients taken to non-stroke centers. Patients whose hospital choices were made by patient/family members had significantly (*p* = 0.003) longer travel times than those for whom the choice was made by EMS personnel. As expected, when the mode to the hospital was RLS, the median travel time (median 19) was significantly (*p* = 0.0001) lower than no RLS mode (median 26).

#### Suspected myocardial infarction (MI)

Summary statistics for the different time intervals for suspected stroke patients are presented in Table 2. Similar to suspected stroke patients, EMS transport times for suspected MI patients differed between the EMS agencies (Table 2). Patients served by EMS 2 had significantly (*p* < 0.0001) shorter response times, but significantly (*p* < 0.0001) longer travel and total times compared to EMS 1 patients. As for stroke patients, it is important to point out that although the absolute difference in response time intervals (2 min) was statistically significant it may not have been large enough to be clinically important. However, the absolute difference in both MI total time interval (7 min) and travel time interval (9 min) were both statistically significant and large enough to be potentially clinically important as an additional 7–9 min in emergency treatment situations would be critical in avoiding complications or fatal outcomes.

EMS 2 female patients had significantly (*p* = 0.002) longer travel times (median: 24; IQR: 7 (20–27)) than males (median: 22; IQR: 10 (19–29)). Study area residents experienced significantly (*p* = 0.002) longer on-scene (median = 14) and travel (median = 24) times than non-residents. Patients whose hospital choice was made by patient/family members had significantly (*p* < 0.0001) longer travel times (median = 25) than those whose choices were made by EMS personnel (median = 22). The use of RLS to the hospital resulted in significantly (*p* = 0.0002) shorter median travel times (median = 21) than when RLS was not used (median = 24).

### Time intervals exceeding guidelines

#### Suspected stroke

Summary statistics for the characteristics of suspected stroke patients whose time intervals exceeded EMS guidelines are should in Table 3. The guidelines for EMS response (≤10 min), on-scene (≤15 min), and total (≤60 min) times were exceeded in 21.5, 34.9, and 2.2 % of suspected stroke cases from EMS 1 and in 17.6, 41.5, and 16.4 % of EMS 2 suspected stroke cases, respectively.

**Table 3:** Characteristics of stroke patients whose time intervals exceeded emergency medical services (EMS) guidelines

	EMS 1			EMS 2		
Time intervals	Total >60 min	Response >10 min	On-scene >15 min	Total >60 min	Response >10 min	On-scene >15 min
	(*n* = 154)	(*n* = 231)	(*n* = 375)	(*n* = 84)	(*n* = 90)	(*n* = 212)
Age						
Median^a^	75	75	77***	64***	63*	58*
Median^b^	74	74	73	53	53	51
Gender: # (%)						
Female	21(91.3)**	-	252 (67.2)	34 (43.6)	-	113 (53.3)
Male	2 (8.7)	-	123 (32.8)	44 (56.4)	-	99 (46.7)
Specialty center: # (%)						
Yes	20 (87)*	-	260 (69.3)	65 (84.4)	-	27 (12.8)*
No	3 (13)	-	115 (30.7)	12 (24.5)	-	184 (87.2)
Season: # (%)						
Fall	7 (30.4)	66 (28.6)	126 (33.6)	31 (39.7)	29 (43.3)*^A^	82 (38.7)
Winter	9 (39.1)	59 (25.5)	101 (26.9)	12 (15.4)	4 (6.0)^B^	39 (18.4)
Spring	2 (8.7)	50 (21.7)	67 (17.9)	15 (19.2)	9 (13.4)^A,B^	32 (15.1)
Summer	5 (31.7)	56 (24.2)	81 (21.6)	20 (35.6)	25 (37.3)^A^	59 (27.8)
Dispatch reason: # (%)						
CVA/Stroke	21 (91.3)	212 (92.6)	338 (90.6)	10 (12.8)	9 (13.4)*^B^	21 (9.9)
Unconscious	0 (0.0)	3 (1.3)	11 (3.0)	11 (14.1)	8 (11.9)^B^	49 (23.1)
Seizure	0 (0.0)	1 (0.5)	2 (0.5)	11 (14.1)	6 (9.0)^B^	29 (13.7)
Fall	0 (0.0)	2 (0.9)	2 (0.5)	7 (9.0)	8 (11.9)^B^	34 (16.0)
Other^c^	2 (8.7)	11 (4.8)	20 (5.4)	39 (50)	36 (53.7)^A^	79 (37.3)

^a^Median age of patients exceeding guidelines

^b^Median age of patients not exceeding guidelines

^c^Other: altered mental status, weakness, dizziness, numbness, headache, unknown

*p ≤0.05

**p ≤ 0.01

***p ≤ 0.001

-Comparisons not performed due to biological implausibility in association between the factors concerned

^A,B,C,D^Categories with different letters are significantly (p < 0.05) different from each other while those with the same letters are not significant different from each

Significantly fewer patients with critical dispatch reasons exceeded the response time guidelines compared to those with “Other” dispatch reasons. Study area residents were significantly more likely to have on-scene times that exceeded guidelines compared to non-residents among EMS 2 suspected stroke patients (Table 4). Significantly more patients, whose hospital choices were made by the patient/family, had total times exceeding guidelines than those for whom EMS personnel chose the hospital.

**Table 4:** Characteristics of patients transported for ^a^EMS 2 whose time intervals exceeded guidelines

	Stroke			Myocardial Infarction		
Time intervals	Total >60 min	Response >10 min	On-scene >15 min	Total >60 min	Response >10 min	On-scene >15 min
	(*n* = 84)	(*n* = 90)	(*n* = 212)	(*n* = 84)	(*n* = 95)	(*n* = 218)
Study area resident: #(%)						
Residents	52 (66.7)*	47 (70.2)**	116 (53.2)*	59 (73.8)**	50 (67.6)	116 (53.2)*
Non-residents	26 (33.3)	20 (29.9)	102 (46.8)	21 (26.3)	24 (32.3)	102 (46.8)
Ambulance mode to scene: # (%)						
Lights/Sirens	72 (92.3)	58 (86.6)	208 (95.4)	77 (96.3)	72 (97.3)	208 (95.4)
No Lights/Sirens	6 (7.7)	9 (13.4)	10 (4.6)	3 (3.75)	2 (2.7)	10 (4.6)
Ambulance mode to hospital: # (%)						
Lights/Sirens	49 (62.8)	24 (35.8)	125 (57)**	52 (65)	28 (37.8)	125 (57) ***
No Lights/Sirens	29 (37.2)	43 (64.2)	93 (43)	28 (35)	46 (62.2)	93 (43)
Hospital choice made by: # (%)						
EMS	30(38)***	47 (70.2)*	160 (73.4)	33 (41.3)***	53 (71.6)	160 (73.4)
Patient/family: # (%)	48 (62)	20 (29.9)	58 (26.6)	47 (58.8)	21 (14.3)	58 (26.6)

*p ≤ 0.05

**p ≤ 0.01

***p ≤ 0.001

^a^ *EMS* Emergency Medical Services

#### Suspected myocardial infarction (MI)

The EMS guidelines for response, on-scene, and total times were exceeded in 18.0, 34.6, and 2.4 % of suspected MI patients from EMS 1 and in 16.5, 37.9, and 14.6 % of EMS 2 patients, respectively. Older patients and those taken to cardiac centers accounted for significantly more of EMS 1 patients that exceeded the on-scene time guideline than younger patients and those not taken to cardiac centers.

Older EMS 2 patients were more likely to have both total and on-scene times that exceeded the guidelines (Table 5). Study area residents were significantly more likely to have total and on-scene times exceeding guidelines than non-residents among EMS 2 suspected MI patients (Table 4). When the hospital choice was made by the patient/family, more patients had total times exceeding guidelines than those whose hospital choice was made by EMS personnel. Patients that had on-scene times exceeding the guidelines were more likely to have had RLS mode to the hospital used (Table 4).

**Table 5:** Characteristics of myocardial infarction cases whose time intervals exceeded emergency medical services guidelines

	EMS 1			EMS 2		
Time intervals	Total >60 min	Response >10 min	On-scene >15 min	Total >60 min	Response >10 min	On-scene >15 min
	(*n* = 42)	(*n* = 315)	(*n* = 606)	(*n* = 84)	(*n* = 95)	(*n* = 218)
Age (median)						
Median^a^	60.5	59	62***	69***	62	62.5***
Median^b^	58	58	56	56	57.5	55
Gender: # (%)						
Female	25 (59.5)	-	335 (55.3)	49 (61.3)	-	116 (53.2)
Male	17 (40.5)	-	271 (44.7)	31 (38.8)	-	102 (46.8)
Specialty center: # (%)						
Yes	3 (7.1)	-	589 (97.2)*	13 (16.3)***	-	11 (5.1)
No	39 (92.9)	-	17 (1.0)	67 (83.8)	-	207 (94.9)
Season: # (%)						
Fall	19 (45.2)	111 (35.3)	255 (42.1)	18 (22.5)	21 (28.4)	64 (29.4)
Winter	7 (16.7)	55 (17.5)	108 (17.8)	21 (26.3)	23 (31.2)	52 (23.9)
Spring	8 (19.1)	71 (22.5)	135 (22.3)	16 (20.0)	12 (16.2)	36 (16.5)
Summer	8 (19.1)	78 (24.8)	108 (17.8)	25 (31.3)	18 (24.3)	66 (30.3)
Dispatch reason: # (%)						
Chest pain	41 (97.6)	310 (98.4)	599 (98.5)	38 (47.5)	36 (48.7)	113 (51.9)^*a^
Short of Breath	0 (0.0)	1 (0.3)	1 (0.2)	24 (30)	18 (24.3)	58 (26.6)^b^
Other^c^	1 (2.4)	4 (1.3)	5 (0.8)	18 (22.5)	20 (27.0)	47 (21.6)^b^

^a^Median age of patients that exceeded the time guideline

^b^Median age of patients that did not exceed the time guideline

^c^ Other dispatch reasons: altered mental status, weakness, dizziness, numbness, headache, unknown

-Comparisons not performed due to biological implausibility in association between the factors concerned

*p ≤ 0.05

**p ≤ 0.01

***p ≤ 0.001

### Logistic model predictors of time intervals exceeding EMS guidelines

#### Suspected myocardial infarction

Results of the multivariable logistic model investigating predictors of time intervals exceeding guidelines for MI patients is shown in Table 6. Based on these results, for every decade increase in the age of the patient, the adjusted odds of total time exceeding guidelines increased by 21 % for patients transported by EMS 2. However, since there was significant effect modification between EMS and age, there was actually a sparing effect of age (lower adjusted odds) for older patients transported by EMS 1. Additionally, patients not taken to a specialty center had a 44 % reduced adjusted odds of having total time exceeding EMS guidelines. It is worth noting that there was significant interaction between age and EMS agency implying that the impact of EMS agency depends on the age of the patient. Response time was significantly associated with age, EMS agency and season. The adjusted odds of response times > 10 min was significantly higher during the fall and summer months compared to the winter. By contrast, these adjusted odds were significantly lower for EMS 1 than 2 and for spring compared to winter season. The model for on-scene times exceeding EMS guidelines showed evidence of significant effect modification between season and EMS implying that the effect of EMS depends on the season of the year and vice versa.

**Table 6:** Logistic model significant predictors of myocardial infarction time intervals exceeding emergency medical services guidelines

Predictors	Adjusted Odds Ratio	*p*-value	95 % Confidence Interval
Outcome: Total Time > 60 Min			
Age (x10 years)	1.212	0.0008	1.083, 1.355
EMS 1	1.093	0.809	0.530, 2.255
2	Referent	Referent	Referent
Specialty Center (No)	0.560	0.0004	0.408, 0.770
(Yes)	Referent	Referent	Referent
EMS X Age (x10 years)	0.843	0.003	0.753,
Outcome: Response Time > 10 Min			
Age (x10 years)	1.112	0.0019	1.040, 1.188
EMS 1	0.839	0.0097	0.733, 0.958
2	Referent	Referent	Referent
EMS X Season (Fall)	1.271	0.0106	1.058, 1.528
EMS X Season (Spring)	0.744	0.0089	0.596, 0.929
EMS X Season (Summer)^1^	1.076	0.499	0.870, 1.328
EMS X Season (Winter)	Referent	Referent	Referent
Outcome: On-Scene Time > 15 Min			
Age (x10 years)	1.186	<0.0001	1.130, 1.246
EMS 1	0.901	0.0445	0.813, 0.998
2	Referent	Referent	Referent
Season (Fall)^b^	1.056	0.4979	0.901, 1.239
Season (Spring)^b^	0.848	0.0870	0.701, 1.024
Season (Summer)^b^	0.981	0.8250	0.825, 1.165
Season (Winter)	Referent	Referent	Referent
EMS X Season (Fall)	1.273	0.0029	1.087, 1.493
EMS X Season (Spring)	1.047	0.6338	0.866, 1.266
EMS X Season (Summer)^a^	0.790	0.0073	0.665, 0.938
EMS X Season (Winter)	Referent	Referent	Referent

^a^Although EMS X Season (summer) was not significant, there was an overall statistical significance (*p* = 0.0136) of the interaction term

^b^Although the main effect of season was not significant in the model, season was kept in the model because of the overall statistical significance (*p* = 0.039) of the EMS X Season interaction term

**Table 7:** Logistic model significant predictors of stroke time intervals exceeding emergency medical services guidelines

Predictors	Coefficient	*p*-value	95 % Confidence Interval
Outcome: Total Time > 60 Min			
EMS 1	0.277	<0.0001	0.205, 0.373
2	Referent	Referent	Referent
Specialty Center (No)	0.710	<0.0174	0.536, 0.942
(Yes)	Referent	Referent	Referent
Outcome: Response Time > 10 Min			
EMS 1^a^	0.885	0.0938	0.767, 1.021
2	Referent	Referent	Referent
Outcome: On-Scene Time > 15 Min			
Age (x10 years)	1.147	<0.0001	1.081, 1.217
EMS 1	0.773	<0.0001	0.685, 0.871
2	Referent	Referent	Referent

^a^None of the variables were significant in the logistic model but EMS was forced in the model because of the *apriori* belief that EMS is a potentially important predictor

#### Suspected stroke

Based on the multivariable logistic regression model, predictors of total time exceeding EMS guidelines were EMS and the patient being taken to specialty center (Table 7) . Patients served by EMS 1 had a 72 % lower adjusted odds of having total time exceeding EMS guidelines compared to those served by EMS 2. Moreover, patients not taken to a specialty center had 29 % lower adjusted odds of having total time exceeding EMS guidelines than those taken to specialty centers. The adjusted odds of response times exceeding 10 min had no significant association with any of the variables assessed in the multivariable logistic model. However, EMS was forced in all models, including this one, because it was one of the main factors under investigation and due to the *a priori* hypothesis that time intervals may be different based on EMS agency/area served. The adjusted odds of on-scene time exceeding EMS guidelines was significantly higher (*p* < 0.0001) for older patients and significantly lower (*p* < 0.0001) for EMS 1. Thus, for every decade increase in age of the patient, there was a 15 % increase in the adjusted odds of on-scene time exceeding EMS guidelines. Additionally, patients served by EMS 1 had a 23 % lower adjusted odds of having on-scene time exceeding EMS guideline than those served by EMS 2.

### Article summary

1. Why is this topic important? Prehospital delays in receiving emergency stroke and myocardial infarction (MI) care have significant clinical impacts on health outcomes. Identification of specific areas/intervals where disparities in Emergency Medical Services (EMS) transport delays occur is critical for guiding health planning to help reduce delays and improve health outcomes.
2. What does this study attempt to show? This study attempts to identify disparities in EMS transport times for suspected stroke and MI patients.
3. What are the key findings? Total transport time exceeding guidelines was influenced by season, EMS agency responsible, patient age and whether or not the patient was transported to a specialty center. Over a third of the calls had on-scene times exceeding the recommended 15 min. Patients served by EMS 2 tended to live in rural communities and experienced significantly (*p* < 0.0001) longer travel times (median = 23 min) than those served by EMS 1 (median = 14.5 min). Transport times exceeding recommended limits tended to involve older patients and those taken to specialty (stroke or cardiac) centers.
4. How is patient care impacted? Since use of EMS are critical for timely access to stroke and MI treatments, the findings of this study are important for guiding local health initiatives that seek to improve health services and outcomes for stroke and MI patients.

## Discussion

A number of past studies investigating prehospital delays treated this delay as one time interval. However, to better identify disparities and target interventions, prehospital delays need to be divided into sub-intervals (i.e. response time, on-scene time, and travel time to the hospital) so as to identify the interval where most delay occurs. Moreover, it is important to identify predictors of these delays so as to guide future studies as well as health planning programs geared towards improving emergency transport of stroke and MI patients. Therefore, the focus of this study was to: (i) identify and describe the disparities in EMS transport delays for suspected MI and stroke patients and (ii) identify predictors of different EMS time interval delays so as to guide future studies and improvement programs. This study addresses this by investigating the predictors of time intervals (response time, on-scene time and total time) not meeting EMS guidelines. It is our belief that the identification of these disparities and their predictors is the first step in addressing the problem of transport delays.

Studies have shown that prehospital delays for suspected stroke and MI patients have a significant impact on eligibility for and timeliness to receive emergency treatments [6]. Although, EMS utilization has been shown to reduce treatment delays for stroke [6, 15] and MI [4] patients, only a few studies have investigated the specific time intervals related to EMS transport as a component of prehospital delay [7–9, 16]. In the current study, the median response times were similar to those from other studies: 5 min [7], 5.5 min [8], 6 min [16], 7.5 min [9] and 8 min [17]. Median on-scene times for the current study were also consistent with those from other studies that reported on-scene times of 13 min for stroke [7] and 14.5 min for MI [9], but lower than others that reported median on-scene times for stroke patients ranging from 18 to 20 min [8, 17]. Based on the multivariable logistic models, the adjusted odds of having EMS times, for suspected stroke and MI, greater than the EMS guidelines were generally significantly lower for EMS 1 than EMS 2. This was the case for all investigated delay times except for response times involving stroke patients. The reason for the shorter response times in EMS 2 than EMS 1 for stroke patients is unclear and is evidently an area that needs further investigations in future studies. However, EMS 2 tended to use RLS quite frequently during travel to the scene and this might have contributed to the shorter response times. However, future studies of EMS protocols will need to be performed to specifically investigate reasons for these differences. It is worth noting that although short on-scene times are not necessarily always best for the patient, especially if the short times are due to omissions of indicated basic and/or advanced procedures [18], other studies have reported that differences in EMS on-scene times may reflect varying levels of efficiency, experience, attitude of EMS personnel serving different populations [7] as well as potential omission of indicated procedures [18]. Unfortunately, since this was a retrospective study using EMS call records, data on specific differences in protocols of the two EMS agencies were not available and therefore the reasons for the observed differences could not be investigated further. Suffice it to say that these differences will have been explained by the EMS variable in the multivariable logistic models. However, future studies will need to investigate more factors (such as EMS protocol differences, call volumes in relation to available equipment and personnel, etc.), in addition to geography, that may help explain the observed differences to help better understand the observed differences in the odds of times exceeding guidelines at the EMS level.

The reason for the comparison of on-scene time across seasons was to assess if there was additional delay during winter resulting from the need to prepare the patients for the colder conditions during winter transport compared to the warmer seasons. While the on-scene time accounted for the longest EMS time component in one study [17], travel time to the hospital comprised the longest time delay in this study. Travel times for EMS 2 are generally longer because of the distribution of hospitals in EMS 2 compared to EMS 1. Generally, the area served by EMS 1 has more hospitals and therefore travel times to the nearest hospital will be shorter. This is evidenced by the fact that EMS 1 tended to have lower adjusted odds of having total time exceeding guidelines compared to EMS 2. Moreover, the areas served by EMS 2 do not have any stroke centers necessitating longer travel times to stroke centers. Unfortunately, data on call locations were not made available to the investigators to maintain anonymity and confidentiality of the patients and therefore distance to the nearest hospital could not be calculated. However, it is interesting to note that the adjusted odds of on-scene time exceeding 10 min was influenced by the age of the patient, season and EMS. Moreover, significant effect modification was noted between EMS and season. This is likely due to the increased traffic during the warner tourism months that potentially results in increased traffic congestion and potential delays during the fall and spring months. The median travel time to the hospital was reported by two studies in urban areas as 11 min for stroke patients [7, 17]. In this study, the travel times for both EMS agencies were longer than 11 min. This is probably related to the distribution of hospitals in urban versus rural areas. In our previous study that included the current study area, we reported longer travel times for some rural areas [19]. Clustering of specialty centers in urban areas, compounds the problem of disparities in access to emergency care for suspected stroke and MI patients [20].

Patient demographic factors reported by some studies to be associated with increased prehospital delay for MI include: older age, females, and black ethnicity [17]. However, the relationships seem to be less clear for suspected stroke patients with some reporting significant associations [7], while others reporting no associations [15]. The observed higher proportion (68 %) of females in the suspected stroke group is unclear but is probably due to the fact that females tend to use the health system more than males. The current study found that both age and gender had significant simple associations with longer delays for some time intervals for both suspected stroke and MI. However, based on the multivariable model, gender was not significantly associated with any of the time delays. Older age had a significant association with all the MI time delays investigated as well as on-scene time delay for stroke patients. The reason for the longer delays associated with older patients may be related to longer times required to stabilize the patients and potential challenges of communication in getting patient history on-scene. It is worth noting that other studies have reported that EMS is more likely to be utilized by older or female patients [21]. Similar to another study [17], males were more likely to have serious dispatch reasons compared to females. These dispatch reasons were also associated with increased use of RLS to the hospital and use of specialty centers, resulting in shorter response, travel, and total times for more critical patients in this study. Similar results for serious symptoms have been reported by other studies [15, 17]. The association between RLS to hospital and response time was investigated to assess if the EMS personnel tended to use RLS to the hospital after realizing the response time to the scene was long and hence attempt to reduce the total time by using RLS in an effort to shorten the time to the hospital. However, there was no association between the two implying this probably did not occur. Race could not be investigated due to lack of variability (96 % white, non-Hispanic) in the population.

Over 40 % of EMS 2 suspected stroke and MI patients were non-residents and despite similarity in patient demographics between study area residents and non-residents, significant differences on delay intervals were observed. For EMS 2, residents were more likely to have longer response times because they were spread throughout the study area, including rural areas, than non-residents that were clustered in the urban areas. Study area residents were more likely to have the hospital chosen by the patient/family member as opposed to EMS, resulting in longer delays for residents than non-residents. Season was investigated in the current study for potential association with transport times due to potential difficult/hazardous road conditions during the winter months especially in the more mountainous areas that present particularly challenging driving conditions during the winter months. Interestingly, longer delays were observed during the fall (not winter season). Similar to findings from another study [7], the longer delays in response time during the fall season probably reflects increased call volumes and traffic congestion due to increased numbers of tourists/visitors to the study area during the fall.

As expected and based on the multivariable logistic model, patients not transported to specialty centers had lower adjusted odds of total time exceeding 60 min compared to those that were transported to specialty centers. This is expected because patients transported to specialty centers (which are few) have to travel longer distances compared to those not taken to such centers. Although patients with high priority dispatch, requiring use of RLS to the hospital, had significant simple association with longer on-scene times, this association disappeared in the multivariable model. However, it should be noted that a previous study reported longer on-scene times for more serious patients and that once at the hospital, these patients were seen by a physician twice as fast [22]. It has been reported that the implications of longer on-scene times are unclear [17]. Other studies have indicated that direct transport to a specialty hospital may not significantly decrease the overall prehospital delay, but that in-hospital delays are significantly reduced and therefore total time to treatment is shorter resulting in better health outcomes [9, 23]. Thus, recommendations for prehospital protocols to incorporate EMS bypassing non-specialty centers are advocated [2, 4].

Based on the simple associations, when the hospital choice was made by the patient/family, the travel time was significantly higher than when EMS personnel made the choice. However, this was not important in the multivariable model. However, it should be pointed out here that the policy observed by both EMS in this study was ‘informed decision’, where the hospital choice of the patient/family must be observed after medical information has been given. When EMS personnel make the decision, the policy is generally to take the patient to the nearest facility with exceptions of level 1 trauma. For the majority of patients in both EMS, the closest hospital was a cardiac center, which would explain the high percentages (96-98 %) of transport to cardiac centers observed. It has been reported that the closest hospital for 40 % of the US population is a cardiac center [24]. However, there were only two specialty stroke centers in the study area. When patients were taken to stroke centers, the decision was more often made by EMS. Given the ‘informed decision’ policies, targeted education encouraging patients/family to choose specialty centers when suspected stroke or MI is suspected would be beneficial. The observed association between age and longer delays has been reported by other studies [7] and may be due to longer time for older patients to get ready for transport. Similar to this study, other studies [25] have also reported significant association between transport delays and seriousness of the condition.

The decision delay component (including recognition of symptoms, decision to seek care, and use of EMS) and the factors affecting it that have been investigated by other studies [16, 21] was beyond the scope of this study which sought to characterize only EMS associated delays. The current study contributes to the body of evidence for only the EMS transport portion of the prehospital delay. Others have suggested, however, that if public education interventions targeted to reduce decision delays are successful over time, delays associated with EMS transport, identified in this study, will become increasingly important [17].

### Limitations

This study was limited by the unavailability of data on the delay before the 911 call as well as data on patient characteristics that may be associated with prehospital delays, including: history of past stroke/MI [17]; co-morbidities [17]; type or severity of the attack, being/living alone, awakening with symptoms, and transfer from another hospital [4, 15]. Furthermore, since all confidential patient data were removed from the database, identified delays for patients could not be linked to their medical records, hospital discharge, or personal outcomes. Future studies should link EMS data to these other databases to investigate impacts of delays on health outcomes.

Although, misclassification of stroke and MI could have occurred since cases were selected based on symptoms, this was unlikely to have had a significant impact on results since the goal was to assess timeliness of EMS transport when MI and stroke are suspected, regardless of final diagnoses. Moreover, a study reported that prehospital times for suspected stroke patients were not different between final diagnoses [8] while another suggested that prehospital times are not likely to be affected by final diagnoses since they are not rendered by EMS but by physicians at hospitals [26].

## Conclusions

In this study, prehospital delays, as measured by total transport time exceeding guidelines was influenced by season, EMS agency responsible, patient age and whether or not the patient was transported to a specialty center. The identified effect modifications between some of the factors imply that their effects should not be considered in isolation. The magnitude of the delays associated with some of the factors are large enough to be clinically important although others, though statistically significant, may not be large enough to be clinically important. Noteworthy was the 72 % lower adjusted odds of total time exceeding guidelines for stroke patients served by EMS 1 compared to those served by EMS 2 implying important geographic disparities. Additionally, patients not taken to specialty centers had 44 % reduced odds of having total time exceeding EMS guidelines a reflection of longer distances and hence transport times to the few available specialty centers. Long transport delays influence time to treatment and hence patient outcomes. Addressing these delays will need to involve strategic telemedicine linkages between hospitals and EMS agencies as well as use of air ambulances. The identified delays and the factors affecting them are vital pieces of information for local health initiatives that seek to improve health services and outcomes for both rural and urban patients. Thus this study’s key contributions are two-fold: (i) it provides insight into different time intervals of prehospital delay for stroke and MI patients beyond what most past studies (that investigated prehospital delay as one time interval) have previously provided; (ii) it provides information to the local EMS agencies to guide their planning since it identified specific time intervals on which guidelines are not yet met to help them identify strategies to improve these time intervals. Moreover, we recommend that these kinds of analyses be performed as part of the routine monitoring of the performance of EMS agencies so as to provide information to guide their planning and service improvement. Suffice it to say that these findings will be useful for guiding future studies and local health initiatives that seek to reduce disparities in prehospital delays so as to improve health services and outcomes for stroke and MI patients.

## Abbreviations

AHA: American heart association
EMS: Emergency medical services
IRB: Institutional review board
JCAHO: Joint commission on the accreditation of health organizations
MI: Myocardial infarction
PCI: Percutaneous coronary intervention
RLS: Red lights/sirens

## References

[1] Roger V, Go A, Lloyd-Jones D, Adams R, Berry J, Brown T (2011). Heart disease and stroke statistics-2011 update a report from the American heart association. Circulation.

[2] Adams H, Del Zoppo G, Alberts M, Bhatt D, Brass L, Furlan A (2007). Guidelines for the early management of adults with ischemic stroke. Circulation.

[3] Em A, Hand M, Pw A, Er B, La G, Lk H (2007). Focused update of the Acc/Aha 2004 guidelines for the management of patients with St-elevation myocardial infarction - a report of the american college of cardiology/american heart association task force on practice guidelines. Circulation 2008.

[4] Ayrik C, Ergene U, Kinay O, Nazli C, Unal B, Ergene O (2006). Factors influencing emergency department arrival time and in-hospital management of patients with acute myocardial infarction. Advances In Therapy.

[5] Moser D, Alberts M, Kimble L, Alonzo A, Croft J, Dracup K (2006). Reducing delay in seeking treatment by patients with acute coronary syndrome and stroke - a scientific statement from the american heart association council on cardiovascular nursing and stroke council. Circulation.

[6] Kr E, Re F, Dl M, Wd R (2009). A comprehensive review of prehospital and in-hospital delay times in acute stroke care. Int J Stroke.

[7] Kleindorfer D, Lindsell C, Broderick J, Flaherty M, Woo D, Ewing I (2006). Community socioeconomic status and prehospital times in acute stroke and transient ischemic attack - Do poorer patients have longer delays from 911 call to the emergency department?. Stroke.

[8] Dunford J, Linnick W, Patel E, Jensen M, Chacon M, Castillo E (2008). Accuracy of stroke recognition by emergency medical dispathers and paramedics - San Diego experience. Prehosp. Emerg. Care.

[9] Studnek J, Garvey L, Blackwell T, Vandeventer S, Ward S (2010). Association between prehospital time intervals and St-elevation myocardial infarction system performance. Circulation.

[10] Warning Signs Of Heart Attack, Stroke & Cardiac Arrest [http://www.heart.org/Heartorg/Conditions/Conditions_Ucm_305346_Subhomepage.Jsp].

[11] Health Care Organization Quality Check [http://www.qualitycheck.org/Consumer/Searchqcr.Aspx].

[12] Sas Institute Inc.: Sas Onlinedoc® 9.2. In*.*, 9.2 Edn. Cary, Nc: Sas Institute Inc.; 2007.

[13] Newgard C, Schmicker Rh H, Trickett J, Davis D, Bulger E, Aufderheide T (2010). Emergency medical services intervals and survival in trauma: assessment of the “golden hour” in a North American prospective cohort. Ann Emerg Med.

[14] Dohoo I, Martin W, Stryhn H (2012). Methods in epidemiologic research.

[15] Inatomi Y, Yonehara T, Hashimoto Y, Hirano T, Uchino M (2008). Pre-hospital delay in the Use of intravenous Rt-Pa for acute ischemic stroke in Japan. J Neurol Sci.

[16] Hutchings C, Mann N, Daya M, Jui J, Goldberg R, Cooper L (2004). Patients with chest pain calling 9-1-1 or self-transporting to reach definitive care: which mode is quicker?. Am Heart J.

[17] Mcginn A, Rosamond W, Goff D, Taylor H, Miles J, Chambless L (2005). Trends in prehospital delay time and Use of emergency medical services for acute myocardial infarction: experience in 4 Us communities from 1987–2000. Am Heart J.

[18] Hawkins S, Morgan S, Waller A, Winslow T, Mccoy M (2001). Effects of ground Ems and Ed personnel on Air medical trauma on-site times. Air Med J.

[19] Busingye D, Pedigo A, Odoi A: Temporal Changes In Geographic Disparities In Access To Emergency Heart Attack And Stroke Care: Are We Any Better Today? Spatial And Spatio-Temporal Epidemiology, In Press, Corrected Proof. 10.1016/j.sste.2011.07.010 22748224

[20] Hippisley-Cox J, Pringle M (2000). Inequalities in access to coronary angiography and revascularisation: the association of deprivation and location of primary care services. British J Gen Prac.

[21] Fussman C, Rafferty A, Lyon-Callo S, Morgenstern L, Reeves M (2010). Lack of association between stroke symptom knowledge and intent to call 911 a population-based survey. Stroke.

[22] Kothari R, Barsan W, Brott T, Broderick J, Ashbrock S (1995). Frequency and accuracy of prehospital diagnosis of acute stroke. Stroke.

[23] Alberts Mj, Latchaw Re, Selman Wr, Shephard T, Hadley Mn, Brass Lm (2005). Recommendations for comprehensive stroke centers: a consensus statement from the Brain Attack Coalition. Stroke.

[24] Nallamothu B, Bates E, Wang Y, Bradley E, Krumholz H (2006). Driving times and distances to hospitals with percutaneous coronary intervention in the united states: implications for prehospital triage of patients with St-elevation myocardial infarction. Circulation.

[25] Teuschl Y, Brainin M (2010). Stroke education: discrepancies among factors influencing prehospital delay and stroke knowledge. Int J Stroke.

[26] Rosamond W, Reeves M, Johnson A, Evenson K, Paul Coverdell Natl Acute S (2006). Documentation of stroke onset time - challenges and recommendations. Am J Prev Med.

